# Supplementary material for: A transdisciplinary, comparative analysis reveals key risks from Arctic permafrost thaw
Source: Commun Earth Environ. 2025 Jan 16;6(1):21. doi: 10.1038/s43247-024-01883-w (PMC11738985; doi:10.1038/s43247-024-01883-w)
Supplement: Supplementary file 2 — Supplementary information (PDF) [file 43247_2024_1883_MOESM2_ESM.pdf]

SUPPLEMENTARY INFORMATION

A transdisciplinary, comparative analysis reveals key risks from Arctic permafrost thaw

Susanna Gartler[1, 2*†], Johanna Scheer[3,4*†], Alexandra Meyer[1, 2*†], Khaled Abass[5, 6], Annett Bartsch[7, 2], Natalia Doloisio[8], Jade Falardeau[9], Gustaf Hugelius[10], Anna Irrgang[11], Jón Haukur Ingimundarson[12], Leneisja Jungsberg[13], Hugues Lantuit[11, 14], Joan Nymand Larsen[12], Rachele Lodi[15,16], Victoria Sophie Martin[17, 2], Louise Mercer[18], David Nielsen[19, 20], Paul Overduin[11], Olga Povoroznyuk[1, 2], Arja Rautio[6], Peter Schweitzer[1, 2], Niek Jesse Speetjens[21, 22], Soňa Tomaškovičová[4], Ulla Timlin[23], Jean-Paul Vanderlinden[24,25], Jorien Vonk[21], Levi Westerveld[26], Thomas Ingeman-Nielsen[4]

[1]Department of Social and Cultural Anthropology, University of Vienna, Vienna, Austria.
[2]Austrian Polar Research Institute (APRI), Vienna, Austria.
[3]Department of Ecology and Environmental Science, Umeå University, Umeå, Sweden.
[4]Department of Environmental & Resource Engineering, Technical University of Denmark, Kongens Lyngby, Denmark.
[5]Department of Environmental Health Sciences, College of Health Sciences, University of Sharjah, Sharjah, United Arab Emirates.
[6]Research Unit of Biomedicine and Internal Medicine, University of Oulu, Oulu, Finland.
[7]b.geos, Korneuburg, Austria.
[8]CEARC Research Center, Université Paris Saclay, Orsay, France.
[9]Geotop Research Center in Earth System Dynamics, Département des sciences de la Terre et de l'atmosphère, Université du Québec à Montréal, Montréal, Canada.
[10]Department of Physical Geography and Bolin Centre for Climate Research, Stockholm University, Stockholm, Sweden.
[11]Permafrost Research Section, Alfred Wegener Institute Helmholtz Centre for Polar and Marine Research, Potsdam, Germany.
[12]Stefansson Arctic Institute and University of Akureyri, Akureyri, Iceland.
[13]Nordregio, Stockholm, Sweden.
[14]Institute of Geosciences, University of Potsdam, Potsdam, Germany.
[15]Institute of Polar Sciences, National Research Council, Venice, Italy.
[16]Department of Environmental Sciences, Informatics and Statistics, Ca' Foscari University of Venice, Venice, Italy.
[17]Centre for Microbiology and Environmental Systems Science, University of Vienna, Vienna, Austria.
[18]Department of Geography and Environmental Sciences, Northumbria University, Newcastle, United Kingdom.
[19]Max Planck Institute for Meteorology, Hamburg, Germany.
[20]Center for Earth System Research and Sustainability, University of Hamburg, Hamburg, Germany.
[21]Department of Earth Sciences, Vrije Universiteit Amsterdam, Amsterdam, Netherlands.

[22]School of Environmental Studies, University of Victoria, Victoria, BC, Canada.
[23]Biomedicine and Internal Medicine, Faculty of Medicine, University of Oulu, Oulu, Finland.
[24]Laboratoire CEARC, Paris Saclay University, Université de Versailles Saint-Quentin-en-Yvelines, Guyancourt, France.
[25]Centre for the Study of the Sciences and the Humanities, University of Bergen, Bergen, Norway.
[26]Grid Arendal, Arendal, Norway.

*Corresponding author(s). E-mail(s): susanna.gartler@gmail.com ; johanna.scheer@protonmail.com; alexandra.meyer@univie.ac.at;
†These authors contributed equally to this work.

This document contains supporting information for the manuscript entitled: A transdisciplinary, comparative analysis reveals key risks from Arctic permafrost thaw. Two main tables provide additional details to the i) primary data collection, and ii) data collection and processing undertaken as part of the risk analysis described in the manuscript Methods. Relevant references are listed at the end of the document.

Supplementary Table 1: Overview of the data collection methods, types and analysis methods undertaken as part of the primary data collection.

Disciplines	Specializations	Data collection methods	Collection period	Data types	Data analysis methods	Selected references
Environmental & physical sciences	Terrestrial permafrost	• Soil layer logging<br>• Ground ice content estimation<br>• Vegetation characterization<br>• Permafrost sediment, soil, water & root sampling	08-2018 & 08-2019	• Temperature & active layer thickness data<br>• Ground ice content estimates<br>• Photos, field notes & coordinates<br>• Soil, sediment & water properties (persistant organic pollutants, microbial communities, etc.)	• Laboratory techniques (incubations, sequencing, mass spectrometry, etc.)<br>• Soil organic carbon & nitrogen stock mapping<br>• Remote sensing techniques (synthetic aperture radar data, digital surface model, etc.)<br>• Geospatial analyses & modelling (dissolved organic carbon fluxes, etc.)<br>• Statistical analyses	A'Campo et al. (2021) [1]<br>Zhang et al. (2022) [2]<br>Speetjens et al. (2022) [3]<br>Lodi (2023) [4]<br>Wagner et al. (2023) [5]
	Coastal permafrost	• River water sampling<br>• Nearshore sediment & water sampling<br>• Soil porewater extraction<br>• Remote sensing discharge measurement	08-2018 & 08-2019	• OM optical properties<br>• Carbon concentration<br>• Carbon & water isotopes<br>• Satellite imagery<br>• Stream discharge<br>• Weather data	• Laboratory techniques (incubation)<br>• Isotope analysis<br>• Statistical analyses<br>• Spatial analysis & modelling	Bartsch et al. (2020) [6]<br>Brhun et al. (2021) [7]<br>Tanksi et al. (2021) [8]<br>Speetjens et al. (2022) [3]<br>Speetjens et al. (2024) [9]
	Subsea permafrost	• Literature & database mapping review	2018	• Legacy industry & public access scientific data with geospatial metadata	• Mapping & classification	Angelopoulos et al. (2020) [10]<br>Wilkenskjeld et al. (2022) [11]<br>Miesner et al. (2023) [12]<br>Westerveld et al. (2023) [13]<br>Creel et al. (2024) [14]
	Coastal waters	• Consultation meetings<br>• Participation to community events<br>• Bottom floor mapping<br>• Marine sediment coring & surface sampling	2018	• Marine core microfossil assemblages<br>• Carbon & oxygen isotope from carbonated shells of microfossils	• Sediment sieving<br>• Counting of microfossils under binocular<br>• Carbonate isotope analysis	Falardeau et al. (2023) [15]<br>Falardeau et al. (2023) [16]<br>Falardeau et al. (2023) [17]<br>Falardeau et al. (2023) [18]
	Modelling	• Earth system modelling, coupling ocean, sea ice, land, atmosphere and biogeochemistry components	Model data spanning 1850-2100	• Gridded geospatial variables (ocean, ice, land, atmosphere & biogeochemistry model components)	• Geospatial analyses<br>• Statistical analyses	Nielsen et al. (2020) [19]<br>Nielsen et al. (2022) [20]<br>Miesner et al. (2023) [12]<br>Nielsen et al. (2024) [21]
Engineering sciences	Infrastructure	• Permafrost & soil sampling<br>• In-situ monitoring of permafrost properties<br>• Geophysical surveys<br>• Infrastructure assessment & mapping<br>• Remote sensing<br>• Archival work<br>• Consultation meetings	2018-2023	• Permafrost & soil properties<br>• Temperature & active layer thickness data<br>• Geophysical data<br>• Photos, field notes & geospatial data<br>• Satellite & drone imagery<br>• Notes & transcripts<br>• Historical borehole logs & infrastructure maintenance costs	• Laboratory techniques (water content, porewater salinity, sieving, ect.)<br>• Numerical & statistical modelling<br>• Digitizing & database creation<br>• Geospatial & statistical analyses<br>• Remote sensing processing (landcover classification, etc.)<br>• Qualitative analyses	Bartsch et al. (2020) [22]<br>Bartsch et al. (2021) [23]<br>Lorentzen et al. (2024) [24]<br>Scheer et al. (2024) [25]<br>Tanguy et al. 2024 [26]
Health sciences	Health & pollution	• Interviews, workshops & discussions<br>• Surveys & questionnaires<br>• Bibliometric & systematic reviews<br>• Contaminant monitoring through human data	2017-2023	• Qualitative interview data<br>• Survey results<br>• Data of infectious diseases in human & wild-life<br>• Human data of environmental pollutants & health outcomes	• Qualitative analysis (thematic content)<br>• Statistical analyses<br>• Quantitative analyses	Abass et al. 2018 [27]<br>Waits et al. (2018) [28]<br>Timlin et al. (2021) [29]<br>Timlin et al. (2022) [30]<br>Timlin et al. (2022) [31]
Social sciences	Natural resources, economy & community planning	• Participant observation<br>• Interviews<br>• Surveys	2019-2023	• Field notes<br>• Transcripts<br>• Questionnaire answers	• Thematic analysis<br>• Iterative grounded theory<br>• Descriptive statistics<br>• Qualitative analysis	Doloisio et al. (2020) [32]<br>Jungsberg et al. (2022) [33]<br>Jungsberg et al. (2022) [34]<br>Ramage et al. (2022) [35]<br>Jungsberg et al. (2023) [36]

Adaptation & mitigation	• Participant observation<br>• Workshops<br>• Consultation meetings<br>• Surveys<br>• Heterogeneous & homogeneous focus groups<br>• Structured, semi-structured, open, narrative, ethnographic & expert interviews	2019-2023	• Audio files & transcripts<br>• Field notes & photos<br>• Survey results	• Grounded theory<br>• Content Analysis<br>• Descriptive statistics	Larsen et al. (2021)[37]<br>Meyer et al. (2022)[38]<br>Povoroznyuk et al. (2023)[39]<br>Meyer et al. (2024)[40]

Supplementary Table 2: Detailed description of the risk analysis steps including participants, data inputs, collection and analysis methods, and outputs. The table supplements the workflow diagram (Fig. 10) from the manuscript.

Time period	Event	Participants	Input	Data collection or analysis methods	Output
September 2021	**RW1**: Risk Workshop 1	Full consortium (engineering, physical, environmental, social and health scientists), guest scientists, and representatives from the study areas	All knowledge gathered during primary data collection on permafrost thaw risks	The workshop was based on three components:<br><br>**A) Structured questionnaire for each scientific discipline:**<br>• Can you identify risks related to permafrost thaw that you are working with?<br>• How would you characterize the uncertainties associated with these risks?<br>• Are these risks a concern to humans? If yes, why?<br>• Where could these risks matter?<br><br>Optional questions:<br>• Do you have any comments?<br>• Which definition of risks do you use?<br><br>**B) Panel group discussion on permafrost thaw risks:** Panel group members consisted of local representatives from the study areas, and members of the consortium with extensive knowledge and experience in the study areas.<br><br>**C) Regional break out groups on permafrost thaw risks, corresponding to the Nunataryuk study regions:** These groups consisted of Nunataryuk scientists and invited local representatives from Yakutiya and the Mackenzie River Delta region.	**O1**: Initial list of identified risks, including physical processes, hazards and societal consequences
March 2021	**IE PP**: Internal Exchanges on physical processes	Lead authors and senior Nunataryuk physical scientist	**O1**	The focus group consultation consisted in filtering and streamlining the initial list of physical processes.	**O2**: Reduced list of physical processes
April 2021	**RW2**: Risk Workshop 2	Full consortium and guest scientists	**O2**	The workshop was based on a digital whiteboard exercise during which participants were grouped according to their specialization. The two following tasks were assigned:<br><br>**Task 1:** Go through the direct impacts of permafrost thaw, identify the most relevant ones, add or delete if needed. Identify which direct impacts from permafrost thaw your scientific discipline is working with.<br><br>**Task 2:** Identify the indirect impacts of permafrost thaw and relate them to their corresponding physical processes if applicable.	**O3**: Refined list of physical processes, hazards and societal consequences (i.e., list of uncategorized basic themes)
June 2021	**FW**: Framework Workshop	Engineering, social and health scientists	**O1**	The workshop was based on brainstorming, group discussions and a drawing exercise focusing on risk assessment methods and definitions.	**O4**: Risk definition<br><br>Risk analysis framework[37] guiding the approach presented in the manuscript
February 2022	**RW3**: Risk Workshop 3	Engineering, social and health scientists	**O1 & O3**	The workshop was based on brainstorming, discussions and thematic network analysis: development of a categorization system (global themes and categories of basic themes).	**O5**: Global themes, i.e., risk components (defined as physical processes and impacts, hazards, societal consequences, perceptions and impacted life domains)<br><br>Set of impacted life domains

March 2022	**IE TNA**: Internal Exchange on Thematic Network Analysis	Lead authors	**O3 & O5**	The thematic network analysis was performed through the identification of physical drivers of and processes resulting from permafrost thaw and ensuing key hazards, and development of a categorization system for the physical processes (categories of basic themes).	**O6**: Categories of basic themes: groups of physical processes and set of five key hazards<br><br>Basic themes categorized according to the global themes and categories of basic themes
May 2022	**RW4**: Risk Workshop 4	Full consortium, graphic designer and representatives from the study areas	**O5 & O6**	The workshop was based on three components:<br><br>A) **Early Career Scientists Workshop:** The relationships between the physical processes, five key hazards and associated societal consequences were printed out and laid on tables. Participants were then asked to comment freely either orally or through written comments on sticky notes.<br><br>B) **Consultation with graphic designer:** Discussions were initiated concerning the visualization of the identified risks and associated relationships between the physical processes, hazards and resulting consequences. The initially identified global themes were merged and simplified.<br><br>C) **Plenary Discussion:** The outcomes of the workshop and discussions were presented and discussed with the full consortium.	**O7**: Revised global themes, i.e., risk components, as physical processes, hazards and societal consequences<br><br>Revised categories of basic themes (groups of physical processes, key hazards, and groups of societal consequences).<br><br>Revised basic themes categorized according to the global themes and categories of basic themes<br><br>First risk graphic visualization
June to November 2022	**CR**: Consortium Ranking	Full consortium and guest scientists	**O7**	The relations between the physical processes and key hazards, and between the key hazards and societal consequences were respectively ranked based on their relevance/importance through focus groups and individual exchanges with relevant consortium scientists.	**O8**: Consortium risk rankings
January to March 2023	**LEE**: Local Expert Evaluation	Local rights- and stakeholders from the three study areas (local researchers, Indigenous and other local representatives)	**O7**	The relations between the key hazards and societal consequences were verified and ranked by local rights- and stakeholders during workshops and meetings as described in Ingeman-Nielsen et al. (2023)[41].	**O9**: Local expert risk rankings
April 2023 to February 2024	**SV**: Synthesis and Visualization	Lead authors and graphic designer	**O7, O8 & O9**	The final risk levels were computed per and across study areas by averaging the consortium and local risk rankings. The visualization of the risk graphic was refined.	**O10**: Composite and local risk graphics

Supplementary References

[1] A'Campo, W., Bartsch, A., Roth, A., Wendleder, A., Martin, V. S. *et al.* Arctic Tundra Land Cover Classification on the Beaufort Coast Using the Kennaugh Element Framework on Dual-Polarimetric TerraSAR-X Imagery. *Remote Sensing* **13** (2021).

[2] Zhang, Z., Fluet-Chouinard, E., Jensen, K., McDonald, K., Hugelius, G. *et al.* Development of the global dataset of Wetland Area and Dynamics for Methane Modeling (WAD2M). *Earth System Science Data* **13**, 2001–2023 (2021).

[3] Speetjens, N. J., Tanski, G., Martin, V., Wagner, J., Richter, A. *et al.* Dissolved organic matter characterization in soils and streams in a small coastal low-Arctic catchment. *Biogeosciences* **19**, 3073–3097 (2022).

[4] Lodi, R. Ptarmigan Bay (Yukon Coast, CA) POPs analysis on permafrost soils (2023). https://doi.org/10.5281/zenodo.8224257.

[5] Wagner, J., Martin, V., Speetjens, N. J., A'Campo, W., Durstewitz, L. *et al.* High resolution mapping shows differences in soil carbon and nitrogen stocks in areas of varying landscape history in Canadian lowland tundra. *Geoderma* **438**, 116652 (2023).

[6] Bartsch, A., Ley, S., Nitze, I., Pointner, G. & Vieira, G. Feasibility Study for the Application of Synthetic Aperture Radar for Coastal Erosion Rate Quantification Across the Arctic. *Frontiers in Environmental Science* **8** (2020).

[7] Bruhn, A. D., Stedmon, C. A., Comte, J., Matsuoka, A., Speetjens, N. J. *et al.* Terrestrial Dissolved Organic Matter Mobilized From Eroding Permafrost Controls Microbial Community Composition and Growth in Arctic Coastal Zones. *Frontiers in Earth Science* **9** (2021).

[8] Tanski, G., Bröder, L., Wagner, D., Knoblauch, C., Lantuit, H. *et al.* Permafrost Carbon and CO2 Pathways Differ at Contrasting Coastal Erosion Sites in the Canadian Arctic. *Frontiers in Earth Science* **9** (2021).

[9] Speetjens, N. J., Berghuijs, W. R., Wagner, J. & Vonk, J. E. Degradation of ice-wedge polygons leads to increased fluxes of water and DOC. *Science of The Total Environment* **920**, 170931 (2024).

[10] Angelopoulos, M., Overduin, P. P., Miesner, F., Grigoriev, M. N. & Vasiliev, A. A. Recent advances in the study of Arctic submarine permafrost. *Permafrost and Periglacial Processes* **31**, 442–453 (2020).

[11] Wilkenskjeld, S., Miesner, F., Overduin, P. P., Puglini, M. & Brovkin, V. Strong increase in thawing of subsea permafrost in the 22nd century caused by anthropogenic climate change. *The Cryosphere* **16**, 1057–1069 (2022).

[12] Miesner, F., Overduin, P. P., Grosse, G., Strauss, J., Langer, M. *et al.* Subsea permafrost organic carbon stocks are large and of dominantly low reactivity. *Scientific reports* **13**, 9425 (2023).

[13] Westerveld, L., Kurvits, T., Schoolmeester, T., Mulelid, O., Eckhoff, T. S. *et al.* *Arctic Permafrost Atlas* (Grid-Arendal, 2023).

[14] Creel, R. C., Miesner, F., Wilkenskjeld, S., Austermann, J. & Overduin, P. P. Glacial isostatic adjustment reduces past and future Arctic subsea permafrost. *Nature Communications* **15**, 3232 (2024).

[15] Falardeau, J., de Vernal, A., Seidenkrantz, M.-S., Fritz, M., Cronin, T. M. *et al.* A 1300-year microfaunal record from the Beaufort Sea shelf indicates exceptional climate-related environmental changes over the last two centuries. *Palaeogeography, Palaeoclimatology, Palaeoecology* **625**, 111670 (2023).

[16] Falardeau, J., de Vernal, A., Fréchette, B., Hillaire-Marcel, C., Archambault, P. *et al.* Impacts of stronger winds and less sea ice on Canadian Beaufort Sea shelf ecosystems since the late 1990s. *Estuarine, Coastal and Shelf Science* **294**, 108520 (2023).

[17] Falardeau, J., de Vernal, A., Seidenkrantz, M.-S., Cronin, T. M., Gemery, L. *et al.* Microfaunal Recording of Recent Environmental Changes in the Herschel Basin, Western Arctic Ocean. *Journal of Foraminiferal Research* **53**, 20–48 (2023).

[18] Falardeau, J., Ouellet-Bernier, M.-M. & Thibodeau Breault, M.-A. Knowledge mobilization in micropaleontology, dendrochronology, historical climate and local knowledge - Education Kit (2023). https://doi.org/10.5281/zenodo.10281640.

[19] Nielsen, D. M., Dobrynin, M., Baehr, J., Razumov, S. & Grigoriev, M. Coastal erosion variability at the southern Laptev Sea linked to winter sea ice and the Arctic Oscillation. *Geophysical Research Letters* **47**, e2019GL086876 (2020).

[20] Nielsen, D. M., Pieper, P., Barkhordarian, A., Overduin, P., Ilyina, T. *et al.* Increase in Arctic coastal erosion and its sensitivity to warming in the twenty-first century. *Nature Climate Change* **12**, 263–270 (2022).

[21] Nielsen, D. M., Chegini, F., Maerz, J., Brune, S., Mathis, M. *et al.* Reduced Arctic Ocean CO2 uptake due to coastal permafrost erosion. *Nature Climate Change* 1–8 (2024).

[22] Bartsch, A., Pointner, G., Ingeman-Nielsen, T. & Lu, W. Towards Circumpolar Mapping of Arctic Settlements and Infrastructure Based on Sentinel-1 and Sentinel-2. *Remote Sensing* **12** (2020).

[23] Bartsch, A., Pointner, G., Nitze, I., Efimova, A., Jakober, D. *et al.* Expanding infrastructure and growing anthropogenic impacts along Arctic coasts. *Environmental Research Letters* **16**, 115013 (2021).

[24] Lorentzen, T. H., Kass, M. A., Scheer, J., Tomaškovičová, S., Christiansen, A. V. *et al.* Exploring the challenges of interpreting near-surface towed transient electromagnetic data on saline permafrost. *GEOPHYSICS* **89**, E113–E128 (2024).

[25] Scheer, J., Tomaškovičová, S. & Ingeman-Nielsen, T. Thaw settlement susceptibility mapping for roads on permafrost - Towards climate-resilient and cost-efficient infrastructure in the Arctic. *Cold Regions Science and Technology* **220**, 104136 (2024).

[26] Tanguy, R. Exposure of Arctic coastal settlements to coastal erosion and permafrost warming. *Authorea Preprints* (2024).

[27] Abass, K., Emelyanova, A. & Rautio, A. Temporal trends of contaminants in Arctic human populations. *Environmental Science and Pollution Research* **25**, 28834–28850 (2018).

[28] Waits, A., Emelyanova, A., Oksanen, A., Abass, K. & Rautio, A. Human infectious diseases and the changing climate in the Arctic. *Environment International* **121**, 703–713 (2018).

[29] Timlin, U., Kauppila, S., Jungsberg, L., Nordström, T., Schmidt-Pedersen, K. *et al.* Perception of Health Challenges, Self-Rated Health and Feeling of Empowerment in a Changing Climate and Environment with Permafrost Thawing. *Sci* **3** (2021).

[30] Timlin, U., Ramage, J., Gartler, S., Nordström, T. & Rautio, A. Self-Rated Health, Life Balance and Feeling of Empowerment When Facing Impacts of Permafrost Thaw—A Case Study from Northern Canada. *Atmosphere* **13** (2022).

[31] Timlin, U., Meyer, A., Nordström, T. & Rautio, A. Permafrost thaw challenges and life in Svalbard. *Current Research in Environmental Sustainability* **4**, 100122 (2022).

[32] Doloisio, N. & Vanderlinden, J.-P. The perception of permafrost thaw in the Sakha Republic (Russia): Narratives, culture and risk in the face of climate change. *Polar Science* **26**, 100589 (2020).

[33] Jungsberg, L., Herslund, L. B., Nilsson, K., Wang, S., Tomaškovičová, S. *et al.* Adaptive capacity to manage permafrost degradation in Northwest Greenland. *Polar Geography* **45**, 58–76 (2022).

[34] Jungsberg, L. Turning Greenland's sand into gold. *Nature Sustainability* **5**, 918–919 (2022).

[35] Justine Ramage, A. M., Leneisja Jungsberg & Gartler, S. 'No longer solid': perceived impacts of permafrost thaw in three Arctic communities. *Polar Geography* **45**, 226–239 (2022).

[36] Jungsberg, L. & Wendt-Lucas, N. ACTIONS, AND RESILIENCE EFFORTS FOR A WARMING CLIMATE IN NORTH GREENLAND. *Routledge Handbook of Climate Change Impacts on Indigenous Peoples and Local*

Communities 358 (2023).

[37] Larsen, J. N., Schweitzer, P., Abass, K., Doloisio, N., Gartler, S. *et al.* Thawing Permafrost in Arctic Coastal Communities: A Framework for Studying Risks from Climate Change. *Sustainability* **13** (2021).

[38] Meyer, A. Physical and feasible: Climate change adaptation in Longyearbyen, Svalbard. *Polar Record* **58**, e29 (2022).

[39] Povoroznyuk, O. & Schweitzer, P. Ignoring environmental change? On fishing quotas and collapsing coastlines in Bykovskiy, Northern Sakha (Yakutiya). *Ambio* **52**, 1211–1220 (2023).

[40] Meyer, A. & Sokolíčková, Z. 'Melting Worlds' and 'Climate Myths': Diverging Stories of Climate Change in Longyearbyen, an Arctic 'Frontline Community'. *Ethnos* **0**, 1–18 (2024).

[41] Ingeman-Nielsen, T., Gartler, S., Meyer, A., Scheer, J., Jungsberg, L. D. M. *et al.* Multidisciplinary Workshops for Early Career Researchers in Arctic Social Sciences and Engineering (2024). https://doi.org/10.5281/zenodo.10556213.
